# Type W Human Endogenous Retrovirus (HERV-W) Integrations and Their Mobilization by L1 Machinery: Contribution to the Human Transcriptome and Impact on the Host Physiopathology

**DOI:** 10.3390/v9070162

**Published:** 2017-06-27

**Authors:** Nicole Grandi, Enzo Tramontano

**Affiliations:** 1 Department of Life and Environmental Sciences, University of Cagliari, Cittadella Universitaria di Monserrato SS554, 09042 Monserrato, Cagliari, Italy; nicole.grandi2@gmail.com; 2 Istituto di Ricerca Genetica e Biomedica, Consiglio Nazionale delle Ricerche (CNR), 09042 Monserrato, Cagliari, Italy

**Keywords:** HERV-W, endogenous retroviruses, Syncytin, autoimmunity, cancer

## Abstract

Human Endogenous Retroviruses (HERVs) are ancient infection relics constituting ~8% of our DNA. While HERVs’ genomic characterization is still ongoing, impressive amounts of data have been obtained regarding their general expression across tissues. Among HERVs, one of the most studied is the W group, which is the sole HERV group specifically mobilized by the long interspersed element-1 (LINE-1) machinery, providing a source of novel insertions by retrotransposition of HERV-W processed pseudogenes, and comprising a member encoding a functional envelope protein coopted for human placentation. The HERV-W group has been intensively investigated for its putative role in several diseases, such as cancer, inflammation, and autoimmunity. Despite major interest in the link between HERV-W expression and human pathogenesis, no conclusive correlation has been demonstrated so far. In general, (i) the absence of a proper identification of the specific HERV-W sequences expressed in a given condition; and (ii) the lack of studies attempting to connect the various observations in the same experimental conditions are the major problems preventing the definitive assessment of the HERV-W impact on human physiopathology. In this review, we summarize the current knowledge on the HERV-W group presence within the human genome and its expression in physiological tissues as well as in the main pathological contexts.

## 1. Introduction

In the last 15 years, great efforts have been made to provide a complete assembled sequence of the human genome, progressively revealing an unexpected, highly repetitive composition. Transposable elements (TEs) account, in fact, for >50% of our genetic material, while protein-coding regions constitute only the ~2% [1]. TEs can be broadly divided in two general classes, based on whether DNA or RNA serves as the intermediate in the process of transposition. Human Endogenous Retroviruses (HERVs) belong to class-I TEs, also called retrotransposons, which are characterized by a RNA intermediate that is reverse-transcribed into a double stranded DNA (dsDNA). This dsDNA, commonly called a provirus, is competent for the subsequent integration into the host cell genome [2]. In addition to HERVs, which have 5′ and 3′ long terminal repeats (LTRs), retrotransposons also comprise elements devoid of LTRs and characterized by 3′ poly(A) repeats that are critical for their retroposition, namely long and short interspersed nuclear elements (LINEs and SINEs, respectively) [3]. 

**Figure 1:**
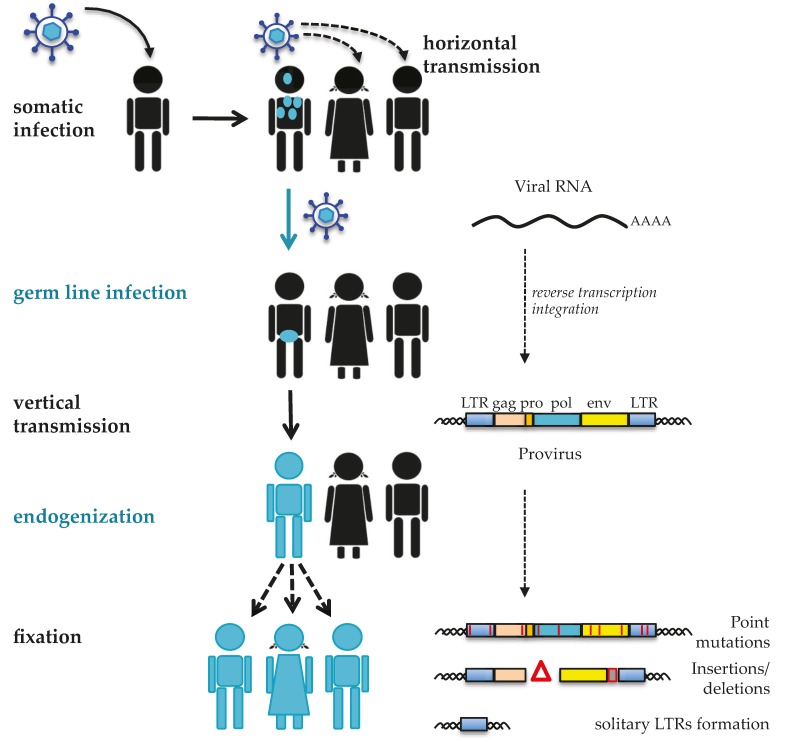
Retrovirus endogenization and human endogenous retroviruses (HERV) formation. During replication, retroviral RNA is reverse-transcribed into a double stranded DNA (dsDNA) provirus and integrated into the cellular genome. All current human retroviruses target somatic cells, showing a horizontal transmission from an infected individual to new hosts. The exogenous retroviruses that gave rise to HERVs were also able to infect germ line cells. In this way, the integrated HERV sequences have been inherited in a Mendelian fashion, being vertically transmitted through the offspring and fixed into the human genome. During evolution, the majority of HERVs accumulated mutations that generally compromised their coding capacity. In several cases, the homologous long terminal repeat (LTR)-LTR recombination has led to the elimination of the whole internal portion, leaving only a solitary LTR as a relic.

HERVs are remnants of ancient retroviral infections acquired by the host genome in several waves that occurred mostly between 100 and 40 million years ago [4]. HERVs were once exogenous retroviruses and, in contrast to the retroviruses currently threatening humans, their infection not only affected somatic cells, but also involved, in particular, the germline. Hence, at the time, the proviral integration into the germline cells’ DNA made HERV sequences stable components of our genome (Figure 1). Such a process of endogenization and the further fixation in the human population have allowed HERVs to be vertically transmitted to offspring in a Mendelian fashion, constituting up to ~8% of the human genome [1]. In general, HERV sequences have been formed by a traditional process of reverse transcription and integration, and thus show a classical proviral structure. The latter is characterized by an internal portion, including the main retroviral genes (*gag*, *pro*, *pol*, and *env*), flanked by the two LTRs. Owing to their long-time persistence in the host genome, however, individual HERV sequences have independently accumulated nucleotide substitutions, deletions, and insertions, often leading to the loss of coding capacity. In several cases, the homologous recombination between the two LTRs of a same provirus led to the elimination of the whole internal portion [5], a phenomenon reflected by the several thousands of solitary LTRs widespread in the human genome.

Despite the abundant presence of HERVs in the human DNA, their general classification at the genomic level has been incomplete and sometimes controversial, due to the increasing amount of bioinformatics data and the concomitant absence of precise taxonomic rules [6]. Based on sequence similarity with respect to the exogenous retroviruses, HERVs were originally divided into three main classes: class I (*Gammaretrovirus*- and *Epsilonretrovirus*-like), class II (*Betaretrovirus*-like), and class III (*Spumaretrovirus*-like). Each class encloses a variable number of HERV groups, which have been named with discordant criteria in over the years, e.g., based on the human tRNA putatively recognized by the primer binding site (PBS) (e.g., HERV-K for Lysine, HERV-W for Tryptophan, and so on) or according to the name of a nearby gene (e.g., HERV-ADP) or a particular amino acid motif (e.g., HERV-FRD). Only very recently, the human genome assembly GRCh37/hg19 has been analyzed with RetroTector program (ReTe) [7], leading to the recognition and global characterization of >3000 HERV insertions [8]. A multi-step classification approach, based on similarity image analysis, *pol* gene phylogeny and taxonomic feature identification, allowed characterization of 39 “canonical” well defined groups of HERVs, and 31 additional “non-canonical” clades [8]. Interestingly, HERV sequences included in the latter showed several degrees of mosaicism that mainly occurred as the consequence of recombination and secondary integration events [8]. Moreover, this comprehensive classification provided a reliable background for the exhaustive characterization of individual HERV groups at the genomic level, which still remains a major genetic and bioinformatics goal [9]. 

In contrast to the genomic characterization, which is still ongoing for most of the HERV groups, there are many studies—mainly based on microarrays, hybridization assays or reverse transcriptase polymerase chain reaction (RT-PCR) approaches—that assessed HERV expression in healthy human tissues and cell lines [10,11,12,13,14,15,16,17]. These reports suggested that HERVs are stable components of the human transcriptome, and display differential expression among the diverse human tissues. In particular, variability of HERV transcription between healthy and pathological samples acted as a driving force to determine HERV’s role in several human disorders, such as cancer, inflammation, autoimmunity, and infectious diseases. Overall, even if the relevance of HERVs expression to the human physiopathological transcriptome is undeniable, its association with the diverse pathological conditions has lacked, until now, sufficient support. In fact, due to the absence of an unequivocal cause-effect relationship between HERV expression and any human disease [18,19,20], a number of studies unfortunately ended in the field of “rumor-virology” [18]. As mentioned above, the failure to establish cause–effect relationships primarily depends on the lack of proper characterization of the HERV single groups at the genomic level. The latter is essential to understand which precise HERV sequence is expressed in a given circumstance [21], and if its expression is beneficial, detrimental, or just functionally linked to a specific condition. It is also important to consider that many of the diseases tentatively linked to HERV expression are chronic conditions with a poorly understood etiology, in which several other factors (either genetic or environmental) could potentially produce a causal association [18]. All these aspects have to be considered in relation to the wide panorama of disparate HERVs expression studies, which includes many disparate data and very few studies attempting to assess the various observations in the proper standardized experimental conditions [18]. Thus, once it is established that HERV transcripts are stable signatures of many pathological conditions, the reliable assessment of their specificity and causality to various diseases will be required to explore HERVs as both etiological contributors and innovative therapeutic targets. 

Among HERVs, the HERV-W group is one of the most intensively investigated for its possible physiopathological effects on the host. After its initial identification as putative causative agent for multiple sclerosis (MS) [22], strong expression of the HERV-W group was found in placental tissues [23]. This observation led to the identification of a single HERV-W member (ERVWE1, locus 7q21.2) still able to encode a functional Envelope (Env) protein, which, during evolution, has been coopted for an important function in placentation [24,25]. On the one hand, this individual HERV-W element and its physiological role have been described in great detail [26,27,28,29,30,31]; on the other hand, the general expression of the HERV-W group has been broadly investigated in a variety of tissues, mainly to find correlations with human diseases. In this way, the HERV-W group hyperexpression has been reported in a great number of pathological contexts, making it one of the most promising endogenous elements to be exploited for novel therapeutic and diagnostic strategies. However, in the great majority of cases, the observed expression profiles were yet not linked to any specific HERV-W sequence, preventing so far a definitive association with human pathology. 

**Figure 2:**
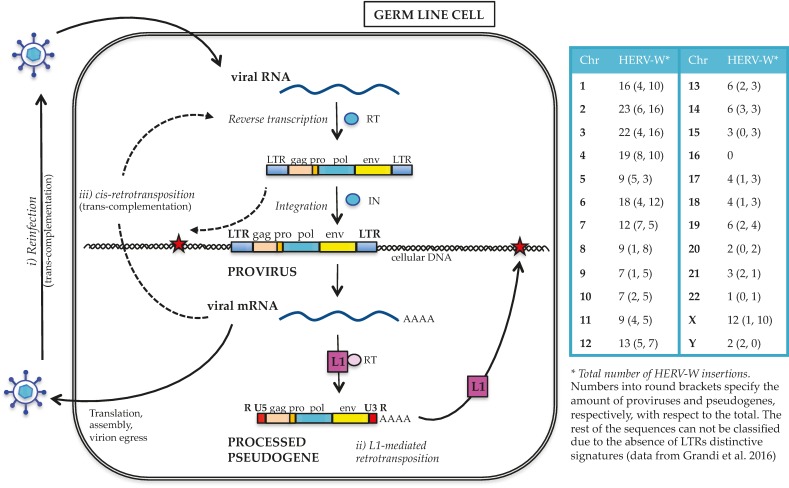
HERV-W sequence amplification in germline cells. The initial acquisition of HERV-W sequences has been due to a traditional retroviral infection process. The viral RNA was reverse transcribed and the proviral dsDNA was integrated into the host cell genome by reverse transcriptase (RT) and integrase (IN) viral enzymes, respectively. Integrated provirus expression provided viral mRNAs, which generated new HERV-W insertions (red stars) through (i) L1-mediated retrotransposition: copy and paste mechanism in which viral mRNAs were reverse-transcribed by L1 RT and inserted into a new genomic position, generating HERV-W processed pseudogenes; (ii) reinfection: proviral mRNAs were translated and the deriving proteins assembled into a mature viral particle, that after its egress could have re-infected the same cell; (iii) *cis*-retrotransposition: HERV-W mRNAs could have been used as templates for further reverse transcription–integration events, leading to the acquisition of new insertions in the absence of an extracellular phase. Owing to the accumulation of mutations over time, the last two mechanisms could have required proteins provided in *trans* by a helper virus. As shown in the table that reports the number of HERV-W insertions in each chromosome, the L1-mediated processed pseudogenes formation was responsible for the acquisition of about the 2/3 of the HERV-W sequences.

It is noteworthy that in contrast to all other known HERV groups, HERV-W transcripts have the unique capacity to be mobilized by LINE-1 (L1) human retrotransposons [32,33]. HERV-W colonization of primate genomes was, in fact, mainly sustained by the L1-mediated formation of processed pseudogenes. This occurred through the reverse transcription of RNA transcripts originating from preexisting HERV-W proviral insertions, and their subsequent integration in new chromosomal positions [32,33] (see Figure 2 and paragraph 2). Considering that the human genome contains about 80–100 L1 elements still competent for retrotransposition [34,35,36], the expression of integrated HERV-W sequences could represent an indirect source of ongoing insertions. This would be more likely to happen in those pathological contexts characterized by an altered epigenetic environment, which could strongly liberate HERV expression, such as cancer and autoimmunity. In this way the general abundance of HERV-W transcripts reported in many tissues could provide a great number of RNA sequences suitable for L1 mobilization, possibly contributing to the intra- and inter-individual genetic variability and being responsible, occasionally, for sporadic insertional mutagenesis and genetic disorders [34,35,36].

The present review focuses on the HERV-W group as an example of the multifaceted effects that retrotransposon movement can exert on the host. In fact, although TEs have been considered as mere genomic parasites for a long time, the presence of such a wide proportion of mobile elements in eukaryotic DNA suggests that they cannot be only detrimental to the host [37]. Hence, we provide a comprehensive overview of the HERV-W group potential impact on human biology, summarizing its contribution to the human genome and the current knowledge of its expression in physiological conditions and, above all, in pathological contexts. We also briefly discuss the current needs for the definitive assessment of the HERV-W expression biological significance, and the future perspectives for its specific exploitation as innovative biomarkers and/or therapeutic targets for a wide range of human diseases. 

## 2. HERV-W Group Contribution to the Human Genome

As for the other HERV groups, HERV-W integration in human germ line cells resulted from traditional retroviral infection (Figure 2). In general, it is still not clear whether the exogenous retrovirus progenitor of HERVs had germ line cells as their specific target or infected such population by chance [38]. In any case, after the entry into germ line cells, the viral RNA was reverse transcribed into proviral dsDNA, flanked by identical LTRs and competent for the insertion into the host cell genome. Repeated integration events determined the initial spread of HERV-W within human chromosomes, with new provirus formation possibly occurring even in the absence of an infectious phase [35] by intracellular reverse transcription and integration of the proviral RNA transcripts [38] (Figure 2). In addition, differently from all other known HERV groups, the HERV-W acquisition by primate genomes has been for the most part sustained by the L1-mediated formation of processed pseudogenes [32,33] (Figure 2). L1 elements encode for a protein with both reverse transcriptase and endonuclease activities [39]. Through these proteins, L1 sequences can copy and paste into new genomic positions not only their RNA, but also the transcripts generated by non-autonomous retrotransposons (*Alu* and SINE–VNTR–*Alu*, or SVA) and by HERV-W proviruses. According to this model, RNA transcripts originating from preexisting HERV-W proviral insertions were reverse transcribed and integrated into additional chromosomal loci by the L1 machinery. These HERV-W processed pseudogenes are characterized by specific signatures that structurally resemble viral mRNA: (i) truncated 5′ and 3′ LTRs, showing a R-U5 and U3-R structure instead of the traditional U3-R-U5 structure, respectively; (ii) a poly(A) tail of variable length, and (iii) a TT/AAAA insertion motif and a 5–15 nucleotides target site duplication [21,32]. As reported in the few studies aimed at characterizing the group at the genomic level [21,32,33], L1-mediated processed pseudogenes acquisition has not been a minor event in the HERV-W diffusion, having formed >2/3 of the group members. The molecular model of L1-mediated HERV-W transcripts retrotransposition, as well as the specific determinants that limited the process to this HERV group, still remain to be clarified.

Three independent studies performed a number of years ago on either isolated human chromosomes [40] or incomplete draft versions of the human genome characterized the HERV-W group at the genomic level [32,33]. Although these studies certainly represent milestones in the analysis of the HERV-W group, the use of different methodologies led to discordant results, currently difficult to retrieve and, especially, to correlate with modern expression data. Recently, a new bioinformatics analysis of the human genome assembly GRCh37/hg19 defined, for the first time, the precise localization and detailed description of 213 HERV-W insertions. The latter were classified, according to their general structure, into proviruses (65), L1-mobilized processed pseudogenes (135), and undefined sequences lacking both LTRs (13) [21]. In this study, the exhaustive characterization of each single HERV-W member in terms of estimated time of integration, genomic context of insertion, and nucleotide sequence provided a dataset that distinguishes the uniqueness of each HERV-W sequence. This dataset is particularly valuable for determining the link between the observed HERV-W expressed products (RNA and proteins) and their specific locus of origin [21]. In addition, some insights regarding the HERV-W group taxonomy were reported for the first time, such as the presence of a second Gag nucleocapsid Zinc finger and the classification of the HERV-W members in two phylogenetic subgroups (named 1 and 2) based on both LTR phylogeny and key mutations [21]. Interestingly, even if the transcripts originated from both HERV-W subgroups were mobilized by the L1 machinery to generate processed pseudogenes, the mechanism was more frequent for subgroup 1 than for subgroup 2 proviruses (1:2.5 and 1:1 ratio, respectively) [21]. The reason for is unclear, but it is possible that the presence of specific sequences made the retrotransposition of subgroup 1 transcripts more efficient or, alternatively, that subgroup 2 elements were expressed at lower levels. Hence, the analysis of the single HERV-W insertions could provide information about L1-mediated HERV-W processed pseudogene formation, which is important for assessing novel retrotransposition events in disease contexts. The molecular elucidation of such events could be important to finally establish the possible pathogenic role of HERV-W processed pseudogenes that, due to their defective structure, have been often disregarded in expression studies, and are thus still poorly investigated in the human physiopathological environment.

In the following sections, we summarize the current knowledge about the HERV-W group expression in both physiological and pathological tissues, based on the many studies performed in the past twenty years. With very few exceptions, these studies investigated the HERV-W group general expression, i.e., without any information on the transcripts genomic origin (specific HERV-W locus) and the structure (provirus or processed pseudogene). If, on the one hand, the available data suggest that HERV-W sequences are differentially expressed in almost all analyzed tissues, being often hyperexpressed in the presence of diseases, on the other hand, these observations deserve more specific investigation aimed at finally identifying which HERV-W loci are selectively deregulated in various conditions. 

## 3. HERV-W Placental Expression and Syncytin-1 Env Protein Cooption for Human Physiology

The reported presence of retroviral particles with reverse transcriptase (RT) activity in MS patients samples [41,42] led to the first description of the so called “MS Retrovirus” (MSRV). Subsequent Southern blot analysis using MSRV-derived probes allowed detection of a previously undescribed HERV multicopy family [22], formally named as HERV-W group [23]. Interestingly, the molecular characterization of the group coding capacity revealed a strong expression restricted to placenta [24] (apart from minor expression in testis [25]), showing the presence of a complete open reading frame (ORF) encoding for two major *env* transcripts (4 and 8 kb) [24,25]. Such ORF was shown to produce a 538 amino acids functional Env protein [23] that was mapped to a HERV-W locus on chromosome 7q21.2 (ERVWE1) [25]. ERVWE1 harbored a 5’LTR functional promoter, exhibiting several binding sites for transcriptional regulators involved in the control of proliferation and differentiation [26,43]. ERVWE1 Env protein was expressed in a panel of different species cell lines, interacting with the type D mammalian retrovirus receptor (hASCT2, human sodium-dependent neutral amino acid transporter type 2) strongly inducing syncytia formation [24,25]. It was therefore named Syncytin-1 [25]. The evidence that syncytia formation could be specifically impaired by Syncytin-1 inhibition (through both specific antibodies [24,25] and anti-sense transcripts [44]) confirmed a central role of this Env protein in the homo- and heterotypic fusogenicity [24,25]. Even if the cell-cell fusion mediated by Syncytin-1 primary depends on its interaction with hASCT2 receptor [24], this Env can efficiently also bind to hASCT1 [45] and even the highly divergent mouse orthologous transporters, mASCT1 and mASCT2, after the elimination of their *N*-linked glycosylation sites [45]. This flexibility indicates a lower restriction of receptor usage as compared to the other retroviral Env proteins, probably due to the strong selective pressure acting on Syncytin-1 throughout evolution [45]. Syncytin-1 placental expression has been specifically confirmed in the villous [25] and extravillous trophoblasts [46], and its strong fusogenic activity has been associated with the formation of the villous syncytiotrophoblast, the main site for trophic exchanges and other placental functions essential for fetal growth and development [24,25]. Beside its central fusogenic role, Syncytin-1 is also directly involved in cytotrophoblast differentiation and proliferation, which is essential for syncytiotrophoblast homeostasis [44,47]. In fact, Syncytin-1 siRNA knockdown in BeWo cultures reduced cell growth and proliferation, probably through cell cycle arrest in G1 phase [47]. In contrast, ectopic overexpression of Syncytin-1 stimulated, as expected, trophoblast proliferation, confirming its critical role in promoting the G1/S transition during syncytiotrophoblast formation, and emphasizing a subtle balance of fusogenic and non-fusogenic functions in the co-regulation of the cytotrophoblast pool [47]. Moreover, cyclic AMP (cAMP), known to regulate cAMP-dependent protein kinases acting in trophoblast fusion and differentiation [48], is also able to control Syncytin-1 promoter [44]. The latter is a bipartite element formed by (i) the ERVWE1 5′LTR, which contains cAMP-responsive elements for placental basal expression; adjacent to (ii) a placenta-restricted cellular enhancer, located within a MaLR (Mammalian apparent LTR retrotrasposon) solitary LTR and acting as URE (Upstream Regulatory Element), to confer high tissue-specific expression [29]. In addition to trophoblast cell-cycle regulation, Syncytin-1 seems to play a role also in the control of trophoblast survival, since the knockdown of its expression in BeWo cells triggered the death pathway mediated by apoptosis-inducing factor (AIF) [49].

In addition to the above mentioned functions in placental morphogenesis and homeostasis, Syncytin-1 was also hypothesized to have a role in maternal immunotolerance to the fetus [24,25,46] through its immunosuppressive domain [25], as previously shown for a murine [50] and a simian [51] retrovirus. Subsequent studies reported the absence of such activity in a mouse model, suggesting a genetic disjunction between fusogenic and immunosuppressive functions (at least in mice) [52]. In human blood, however, Syncytin-1 was effectively able to inhibit the production of Th1 cytokines known to be important modulators of several immunological functions. This suggests a possible role of Syncytin-1 in mediating the shift from cytokine Th1 to Th2 observed during pregnancy that may also contribute to immunomodulation of the maternal system [53]. 

## 4. General HERV-W Expression in Healthy Tissues Other than the Placenta

The Syncytin-1 locus is exceptional, since it retains a residual protein-coding capacity, while the great majority of HERV sequences have accumulated mutations affecting protein production. For this reason, and due to their multi-copy nature, HERVs have often been disregarded in large-scale expression studies and, consequently, have not been exhaustively characterized in terms of functional significance [54]. A number of studies, however, investigated their expression across human tissues and cells. Overall, the results show that the various HERV groups display differential global expression profiles, which could be tissue/cell type-specific and vary depending on tissue state changes (e.g., differentiation, pathogenesis) as well as on environmental and individual conditions. 

As stated above, the HERV-W group is strongly expressed in normal placenta [23,24,25] and shows significant transcriptional activity in testis [25]. In addition, HERV-W transcription in healthy tissues has been monitored using RT-PCR protocols amplifying *gag*, *pol* or *env* genes with primers that, in general, were specifically designed for the placental Syncytin-1 ERVWE1 locus (Table 1). A few other studies investigated expressed sequence tags (ESTs) databases using Syncytin-1 proviral sequence as a query, or analyzed group expression using *pol* probes. In this way, general HERV-W expression has been detected in various human cell lines and healthy tissues—often lacking, however, any information about the transcript’s origin from specific loci (Table 1).

In summary, global HERV-W transcriptional activity in healthy conditions was reported by at least one study in the brain, breast, skeletal muscle, spleen, lungs, digestive trait (stomach, liver, colon), genitourinary apparatus (ovary, endometrium, uterus, prostate, testis, and kidneys), and cardiovascular system (heart, whole blood, peripheral blood mononuclear cells (PBMC)). It is noteworthy that all these reports assessed the HERV-W group generic expression, i.e., without connecting the observed transcripts to a specific locus. Moreover, significant biases could derive from the use of Syncytin-1 provirus and/or MSRV cDNA clone sequences as a query and for the design of primers and probes. This could lead, in fact, to the lack of detection of HERV-W expressed loci with divergent nucleotide sequence as compared to the query, or defective for the single genes analyzed. Another possible bias is due to the potential contamination with genomic DNA, possibly representing a further complication in the analysis of multicopy repetitive elements, if not prevented through a correct treatment of RNA samples, e.g., with DNase. Finally, in the majority of cases, no information on the full-length HERV-W sequences expression and the LTR residual regulatory activity are available, and the samples are often limited in number and sometimes incompletely characterized for the individual’s health status. 

An attempt to connect HERV transcriptome to specific loci of origin was performed by Pérot et al. through a dedicated microarray designed on a collection of >5500 HERVs (including both proviruses and solitary LTRs) that could be reasonably allocated to unique genomic loci [15] (Table 2). Based on their results, the HERV-W group showed, as expected, predominant expression in placenta and testis, attributable to Syncytin-1 locus activity. In addition, five other specific HERV-W loci (one provirus, one processed pseudogene, and three solitary LTRs) were also transcribed in the same tissues, showing in two cases a concomitant LTR promoter activity [15]. Despite the fact that the tissues considered by Pérot et al. were limited (colon, lung, breast, ovary, prostate, testis, uterus and placenta) and that all expressed HERVs are co-localized within human genes that could influence their transcription, the analysis is a remarkable effort to match HERV transcriptome to its specific genomic contributors, taking into account relevant aspects such as promoter activity and tissue specificity.

## 5. Syncytin-1 Expression in Placental Pathologies

Consistent with its proven role in human placentation, Syncytin-1 abnormal expression has been observed in various pathological conditions affecting placental and maternal-fetal physiology, i.e., Pre-eclampsia (PE); hemolysis elevated liver enzymes and low platelet count (HELLP) syndrome; Trisomy 21; intrauterine growth restriction (IUGR) and endometriosis. The main findings in these pathological contexts are summarized below and in the Supplementary Materials (Table S1).

In general, it is worth noting that in many of the diseases affecting placental tissues, hypoxia is a common pathological trait able to influence Syncytin-1 expression (Table S1). In light of this, Syncytin-1 downregulation, commonly observed in diseased placentas, and the consequent reduction in trophoblasts fusion and differentiation, is likely to result from the pathological hypoxic environment. 

PE is a multisystem condition affecting ~5% of pregnant women [60]. It is clinically characterized by hypertension, proteinuria and hypoxia, and is associated with adverse perinatal outcome and preterm birth. A significant fusion reduction in trophoblast cells isolated from PE placentas was reported [61] and, in line with this, the placentas of women affected by PE showed a marked decrease in Syncytin-1 expression [61,62,63,64,65]. Such reduction seems to be correlated with PE severity [61] and depends on Syncytin-1 promoter hypermethylation [65], leading to a consequent decrease in cytotrophoblast differentiation [43]. 

Similar Syncytin-1 expression reduction was found in HELLP syndrome [62,63]. Considering that experimental hypoxia reduces Syncytin-1 expression by 80% in BeWo cells in vitro and in isolated placental cotyledons ex vivo [66], it has been suggested that such reduction in Syncytin-1 expression might arise due to the HELLP failure in trophoblasts arterial transformation and the consequent poor placental perfusion [67]. 

Reduced Syncytin-1 expression was also observed in trophoblast cells from placentas bearing a trisomy 21 fetus. Trophoblast cells were still able to aggregate, but fused poorly or late in culture [68,69,70], and showed increased levels of superoxide dismutase encoded on chromosome 21 [71]. When this antioxidant enzyme was overexpressed in normal cytotrophoblasts, impairment in syncytiotrophoblast formation as well as abnormal cell fusion and Syncytin-1 downregulation were observed, further suggesting that oxidative states are able to influence trophoblasts Syncytin-1 production [68,71]. Since it is known that hypoxia can activate the caspase apoptotic pathway, the hypoxic environments common to many placental diseases could possibly lead to trophoblast cell death via both this mechanism and the above mentioned AIF pathway [47], specifically triggered by Syncytin-1 decreased expression [47,60]. 

IUGR is another important cause of perinatal morbidity and mortality for both mother and fetus, and it is also related to hypoxia and abnormal trophoblast development. In line with this, IUGR placentas showed significantly lower Syncytin-1 RNA and protein amounts with respect to control placentas [64,72], although still sufficient to mediate trophoblast cells fusion [72]. 

Finally, two studies reported a high HERV-W expression in endometriotic tissues, even though no great differences were found with respect to control tissues [14,57]. This Syncytin-1 upregulation, dependent on the hypomethylation of its promoter, has been proposed to be involved in endometriotic lesion development [73]. 

Overall, these findings have confirmed a pivotal role of Syncytin-1 expression in placental physiology, and showed how its deregulation could contribute to maternal systemic disorders [60].

## 6. HERV-W Expression in Tumorigenesis and Cancer Progression

**Figure 3:**
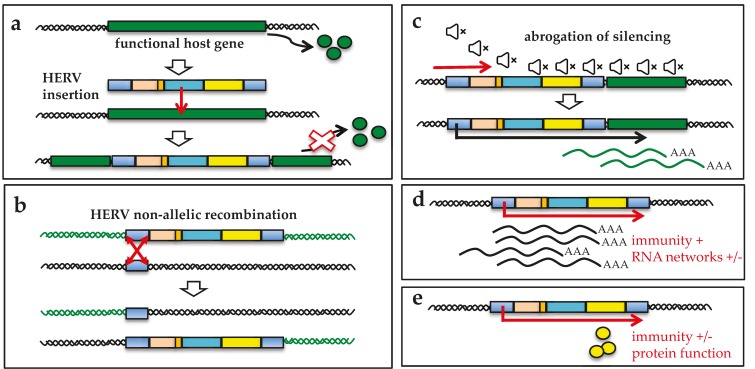
Potential mechanisms of HERV-mediated transformation in tumorigenesis. (**a**) Insertional mutagenesis could disrupt/deregulate host genes; (**b**) non-allelic homologous recombination could induce chromosomal rearrangements; (**c**) transcriptional silencing abrogation could trigger LTR promoter activity; (**d**) accumulation of replication intermediates could evoke immunity and/or deregulate RNA networking; (**e**) protein production could evoke immunity and/or provide oncogenic functions.

Tumorigenesis is a complex multistep process involving both inherited and environmental factors and possible association with HERV expression. Of course, this link has been greatly sustained by the well-described transforming nature of exogenous animal retroviruses, which were originally designated as “RNA tumor viruses”. However, the high copy number and repetitive nature of HERVs may also trigger additional tumorigenesis mechanisms that do not require the production of infectious viral particles, as summarized in Figure 3. In particular, HERV mobilization and integration could be responsible for insertional mutagenesis events (panel a), which could disrupt or deregulate host genes (e.g., oncosuppressors, transcriptional regulators). The presence of repetitive elements could also trigger chromosomal rearrangements by non-allelic homologous recombination (panel b). HERV transcriptional de-repression, possibly prompted by the altered epigenetic environment commonly associated with cancerous tissues, can lead to uncontrolled activation of downstream cellular genes (e.g., oncogenes, transcription factors) (panel c). Even in the absence of protein production, HERVs transcription could stimulate the accumulation of incomplete replication intermediates, which can activate innate immunity pathways and deregulate non-coding RNA networks (panel d). Finally, if a HERV protein is produced, its activities (e.g., fusogenic and/or immunosuppressive functions) and/or abilities (e.g., interaction with cellular proteins) may contribute to tumor development (panel e).

Remarkably, despite several studies that reported the general increase—or even the de novo appearance—of HERV-W transcripts in tumors as compared to healthy tissues (Table 3), it is yet to be understood whether such HERV overexpression is the cause or just a consequence of transformation. In fact, HERV expression is generally silenced by epigenetic mechanisms in normal cells, yet abnormal hypomethylation of CpG dinucleotides is commonly observed in tumor cells. This dysregulation could possibly lead to increased levels of HERV expression as an indirect product of the altered epigenetic environment, instead of a main determinant of the disease onset. Unfortunately, despite the central role of epigenetic changes in influencing HERV transcription, very few studies to date have analyzed the HERV-W sequences methylation status in tumor tissues. One study investigating the epigenetic state of L1 and HERV-W sequences in human ovarian carcinomas reported a consistent reduction in promoter methylation, corresponding to an increase in expression [74]. Upregulation of L1 and HERV-W expression could contribute to tumor progression by de novo mobilization of the abundant HERV-W transcripts. Interestingly, despite an overall increase in hypomethylation, some L1 and HERV-W sequences remained hypermethlyated in malignant samples [74]. This result raises the possibility of more specific regulation of HERV expression leading to a beneficial or detrimental effect on disease progression. HERV-W transcriptional increase in ovarian carcinomas has been reported by Hu et al., but a similar expression level was similarly observed in healthy ovaries, and the number of samples was too low to be statistically significant [57]. HERV-W expression has also been investigated in endometrial carcinomas, due to the presence of giant syncytial cells possibly mediated by Env fusogenic activity. Results showed that Syncytin-1 was upregulated in both benign and malignant tissues; however, the highest expression was detected in endometrial carcinomas [75]. 

In contrast to the above mentioned studies that reported an increase of HERV-W expression in tumor tissues, other studies reported no significant upregulation of HERV-W transcriptional activity in human cancers. Stauffer et al. investigated HERV-W expression in placenta, breast, colon, and kidney cancers, observing that the HERV-W transcription levels in healthy breast and placenta were higher than in corresponding tumor samples [11]. Similarly, Kim et al. reported no significant differences in HERV-W expression between paired tumor and normal adjacent tissues from breast, colon, liver, stomach, and uterus [56].

In addition to the analyses performed on tumor samples from patients, a number of studies investigated the HERV-W group transcriptional activity in cancer cell lines. These studies, however, could not reliably measure the HERV-W expression in cancers, showing a lack of correlation between the expression levels observed in normal tissues and the corresponding cancer cell lines [55]. Moreover, the observed upregulation of HERV-W expression could be, at least in part, a consequence of the tumor cell line environment instead of a specific signature of cancer. For instance, HERV-W RNA levels were increased in three neuroblastoma cell lines (SH-SY5Y, SK-N-DZ, and SK-N-AS), with a selective upregulation during hypoxia recovery and after the treatment with demethylating agents. Both treatments are known to influence HERV transcription with no specificity for tumor environment [78]. Similarly, Díaz-Caballo et al. reported a HERV-W hyperexpression in HCT8 colon carcinoma cells, and proposed a correlation with the induction of a chemotherapy-refractory state [77]. Such increased transcription, however, is possibly the consequence of the experimental induction of a cytostatic stress. 

Studies performed in additional cancer cell lines reported more specific effects associated with HERV-W hyperexpression. SH-SY5Y and another neuroblastoma cell line transfected with HERV-W *env* resulted in increased expression of SK3 (small conductance Ca^2+^-activated K^+^ channel protein 3), an ion channel relevant for neuronal excitotoxicity and linked to various diseases of the nervous system [79]. Such upregulation was proposed to depend on the activation of the SK3 promoter cAMP responsive elements (CRE) as direct consequence of the HERV-W Env-mediated increased phosphorylation of the activating transcription factor CREB (CRE-binding protein) [79]. Similarly, Bjerrgarden et al. hypothesized a direct role of Syncytin-1 fusogenic activity in breast cancer based on the fact that MCF-7 and MDA-MB-231 cells express Syncytin-1 on the cell surface and hence are able to fuse with endothelial cells presenting hASCT-2 receptor [76].

As previously described for HERV-W physiological expression, many reports assessed the altered HERV-W transcription in different tumor tissues, however, very few studies attempted to connect transcription to specific HERV-W loci (Table 4). These studies, even if not conclusive for the definitive association of HERV-W expression to tumor development, provide a more reliable picture of the single HERV-W elements upregulated in different human cancers, and suggest further investigations are warranted to determine HERV-W’s epigenetic status and specific roles in pathogenesis. Moreover, the identification of specific HERV-W loci expressed in cancer tissues also allows evaluation of their structural characteristics. It is interesting to note that, besides 9 HERV-W proviruses, a number of L1-generated processed pseudogenes (6) and solitary LTRs (8) are specifically upregulated in cancer tissues (Table 4). This suggests that highly defective HERV-W elements, especially in the presence of an altered epigenetic control, can be actively transcribed and differentially expressed in cancerous tissues, possibly contributing to the disease progression. 

In particular, the majority of studies reported HERV-W sequence specific expression both in tumoral testis, along with the previously reported Syncytin-1 expression [25], and in a number of other cancers mostly affecting the genitourinary trait. Pérot and coworkers have analyzed paired normal and tumoral tissues through a dedicated microarray (see also paragraph 4 and Table 2), and reported a number of HERV-W loci that were differentially expressed in testis (16), prostate (2) and ovary (1) cancer samples [15]. Similarly, Gimenez and coworkers identified six HERV-W loci, including Syncytin-1, whose expression was upregulated in testicular cancer [80]. The precise localization of these expressed HERV-W sequences allowed comparison of their epigenetic status in normal and tumoral tissues, revealing, in the latter a U3 promoters hypomethylation in at least five out of the six loci [80]. As is the case for ovarian cancer [74], some sequences remained unmethylated in the tumor environments but not in the normal counterparts [80], suggesting the presence of different levels of HERV transcriptional control. When considering bladder urothelial cell carcinomas, Syncytin-1 was significantly hyperexpressed in >75% of the analyzed tumor tissues (*n* = 82) as compared to the 6% of the matched adjacent tissues, increasing proliferation and viability of human immortalized uroepithelial cells [82]. In this case, the identification of specific HERV-W sequences significantly upregulated in tumor tissues also allowed detection of single nucleotide substitutions. The latter were found in positions 142 and 277 of the Syncytin-1 3′LTR, in ~88% tumoral tissues while they were observed only in a small proportion (~5%) of healthy controls. Interestingly, the T142C mutation apparently resulted in selective binding of the c-Myb transcription factor to ERVWE1 LTR, and was possibly associated with the selective enhancement of Syncytin-1 promoter activity in bladder urothelial cell carcinoma [82]. In addition, the expression of specific HERV-W loci has been assessed in mycosis fungoides, the most common Cutaneous T-Cell Lymphoma (CTCL) [81]. In fact, two HERV-W loci in chromosomes 6 (6q21) and 7 (7q21.2—Sincytin-1), which frequently harbor abnormalities and rearrangements in CTCL, were predominantly and significantly upregulated in mycosis fungoides lesions as compared to the same patient intact skin [81].

Despite the number of studies investigating HERV-W expression (either general or associated with specific loci), no human cancer has been unequivocally related to this or any other HERV group. This greatly depends on the lack of definitive evidence that specific HERV sequences are effectively able to induce tumors through the so far proposed mechanisms. Although HERV expression in tumors may contribute to the disease’s clinical outcome, the currently available results suggest only that the HERV-W group has variable expression profiles in both normal and cancerous tissues [11]. As in previous cases, the use of different experimental approaches often affected by potential methodological biases, together with the lack of connection between the observed transcripts and the specific originating locus, currently impedes effective assessment of the biological significance of the HERV-W group expression in tumors. Moreover, the current lack of exhaustive information on HERV-W loci basal expression in healthy tissues clearly limits complete evaluation of their effective dysregulation in the corresponding tumors, which are further complicated by an altered epigenetic regulation. In light of this, even with clear evidence of differential HERV-W expression between tumoral and healthy tissues, further studies are needed to establish which HERV-W loci are actively involved in tumorigenesis and which ones constitute an “epiphenomenon” due to the altered tumoral environment [83].

## 7. HERV-W Expression in MS and Other Autoimmune Diseases

**Figure 4:**
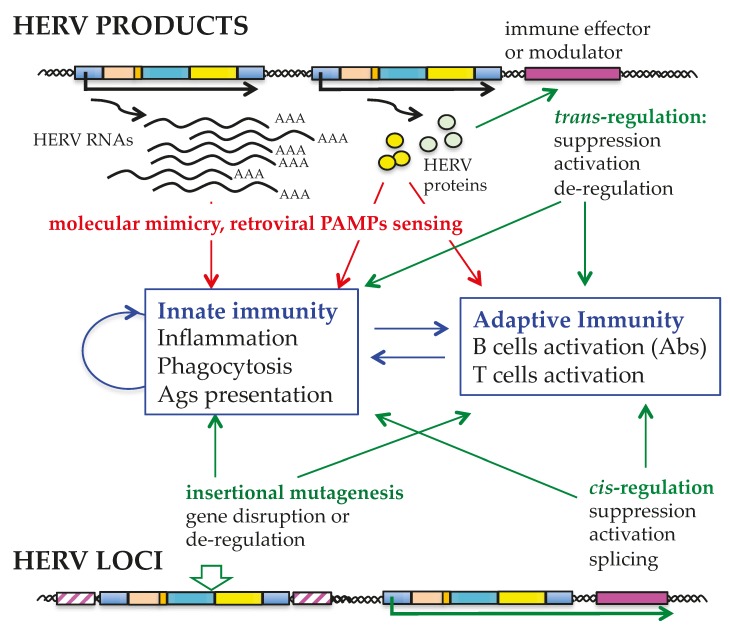
Potential mechanisms of HERV contribution to autoimmunity. HERVs can trigger autoimmunity through the direct sensing of their expression products by pathogen recognition receptors (PRRs) (red) as well as by mediating the deregulation of the host immune effectors and modulators (green). In both cases, the eventual hypomethylated status associated with autoimmunity can upregulate HERVs that are normally silenced in healthy tissues. HERV expressed RNAs and proteins (upper part) can act as pathogen associated molecular patterns (PAMPs) prompting the innate immunity effectors, and, consequently, evoking an adaptive response. HERV proteins can either act as super antigens Ags activating a polyclonal expansion of autoreactive T cells, or deregulate immunity genes. These mechanisms can be also based on the molecular mimicry of HERVs products, due to their identity with the exogenous elements. HERV integrated sequences, or even their sole LTR, (lower part) can affect the host immunity even in the absence of any expressed product, if their insertion disrupts or deregulates genes involved in immune response and its control.

Autoimmune diseases comprise a heterogeneous group of complex multifactorial disorders, all sharing the incorrect recognition of healthy cells and/or the loss of immune tolerance to self-Antigens (Ags) by the immune system. Clinically, such loss of tolerance leads to Antibody (Abs) production and/or cytotoxic T cells responses against body components, resulting in chronic inflammation and tissue destruction. A role for HERV in autoimmune disorders was primarily suggested by (i) the presence of retroviral Ags and/or specific Abs at the site of disease and in patients’ sera, respectively [84,85]; and (ii) an increased HERV expression in patients with autoimmune disorders as compared to healthy individuals [86]. Theoretically, given that HERVs are stable components of the human genome, the immune tolerance to them should have been established during development. Despite this, HERVs still show the ability to induce, or at least to influence, both innate and adaptive immunity [84,85,86,87,88,89] (Figure 4). Currently, the most accepted theory is that HERV expression can evoke autoimmunity by molecular mimicry between common auto-Ags and exogenous retroviral proteins [86,90,91,92,93]. HERV RNAs and proteins may, in fact, be recognized as PAMPs (Pathogen Associated Molecular Patterns) by innate immunity pathogen recognition receptors (PRRs) (recently reviewed in [89]), that determine inflammation and auto-Ab production. Moreover, HERV proteins may act as super-Ags, triggering the non-specific polyclonal activation of auto-reactive T lymphocytes and inducing massive cytokine release. Besides the direct immunogenic effects of retroviral products, HERV proteins may affect the host immune response in additional ways, such as by *trans*-activating/suppressing genes involved in immune modulation. Even in the absence of any expressed product, the mere presence of HERVs can contribute to autoimmunity through insertional mutagenesis events and/or *cis*-regulation of adjacent immune regulatory gene expression at the transcriptional and post-transcriptional level. 

Importantly, as described for cancer, autoimmunity is also influenced by abnormal hypomethylation, which can eventually release HERVs expression [94]. Such an occurrence, even as the mere consequence of epigenetic alterations, could contribute to immunopathogenesis by providing nucleic acids or proteins acting as PAMPs. Furthermore, the loss of epigenetic control can provide HERV-W transcripts suitable for de novo mobilization by L1. Therefore, the proper identification and characterization of the expressed HERV loci is essential to assess their effective involvement in the disease onset and progression.

Focusing now on the HERV-W group, the major field of investigation in autoimmunity is certainly MS, although few studies have been reported for other autoimmune or immune-related disorders. 

### 7.1. Multiple Sclerosis

MS is an autoimmune disorder with poorly understood etiology, and characterized by progressive demyelination of the central nervous system (CNS). Both innate and adaptive immunity dysregulation contributes to MS immunopathogenesis, although adaptive immunity may predominate in the disease onset with selective T and B cell activation accompanying clinical relapses [95]. The precise causes of axon demyelination and damage remain unclear, even if inflammatory molecules such as cytokines, chemokines, prostaglandins, reactive oxygen species and matrix metalloproteinases contribute to MS [95]. In addition, different infectious agents have been investigated for a possible association with MS [95,96,97,98,99,100,101].

As previously mentioned, the HERV-W group was initially related to MS due to its nucleotide identity with MSRV [102,103], a putative retroviral element detected in some MS patients [22,41,42,104,105], and proposed as an exogenous competent member of the HERV-W group [22,106,107,108,109,110]. The origin of MSRV is, however, still highly debated [18,111,112], and recent findings suggest that the previously identified MSRV sequences could have arisen from the expression of a single HERV-W locus, or the in vitro recombination of many HERV-W transcripts [21,113]. In the last twenty years, many studies investigated the HERV-W/MSRV involvement in MS, mainly by (i) the detection of HERV-W/MSRV nucleic acids in MS samples; (ii) the presence of HERV-W/MSRV Ags in MS lesions; (iii) the onset of an immune response against these elements; and (iv) the use of some animal models of MS. Even if all these types of investigation are taken into account, the evidence strongly suggests that the presence of HERV-W/MSRV sequences (i) and Ags (ii) could contribute to a higher prevalence in MS. The clear immunopathogenic potential of these HERV products on cellular-mediated immunity, as shown in both humans (iii) and animal models (iv), could indeed take part, together with other individual factors, to cause MS disease.

#### 7.1.1. Detection of HERV-W/MSRV Nucleic Acids in MS Samples

Regarding the presence of HERV-W/MSRV nucleic acids, most studies focused on the detection of expressed HERV-W/MSRV RNA transcripts, while a few of them investigated the differential amounts of integrated DNA sequences copy-number in MS samples.

HERV-W/MSRV *pol* RNA sequences have been detected by RT-PCR approaches in MS patient brain [114], leptomeningeal, choroid plexus and B cells [22], peripheral blood lymphocytes [115], cerebrospinal fluid (CSF) [22,115,116], serum [116,117] and plasma [115]. Overall, HERV-W/MSRV *pol* amplicons were found in a variable proportion of the MS samples (~50 to 100%) as well as in some healthy controls (0–50%) and non-MS pathological samples (0–65%). These results suggest that HERV-W expression may be associated with the pathological environment and have a role in a particular subset of susceptible individuals. Unfortunately, the expressed RNA sequences were not attributable to specific HERV-W loci [97]. Also MRSV/HERV-W *env* RNA expression was reported to be upregulated in MS patient brains [118,119] and PBMC [120]. Finally, a significantly higher accumulation of both HERV-W/MSRV *pol* and *env* RNAs was reported in MS brains [107] and CSF [121] with respect to healthy and pathological controls, even if all samples tested contained the HERV-W/MSRV transcripts regardless of health/disease status. 

HERV-W/MSRV DNA copy-number was reported to increase in PBMCs of MS patients as compared to controls, and copy number also correlated with disease severity [110,120]. Considering that active HERV-W proliferation ended several millions of years ago, before the evolutionarily speciation of humans [21], it is unlikely that such variation could depend on the presence of unfixed proviral integrations in the modern population, as shown for younger HERV groups. It is indeed more probable that, as described above, the additional HERV-W copies found in MS patients could be due to processed pseudogenes derived from novel L1-mediated retrotransposition events triggered by the autoimmune hypomethylated environment. A positive relationship between HERV-W/MSRV DNA copy number and female gender has been also hypothesized, which is consistent with the higher incidence of MS in women. In particular, the one proviral copy and 10 L1-generated processed pseudogenes of HERV-W on the X chromosome could possibly play a role in MS sex-based variants, similarly to other X chromosome abnormalities [110]. Finally, MSRV *pol* sequences have been detected by fluorescence in situ hybridization (FISH) in the peripheral blood cell DNA from all patients with active MS and healthy controls tested, which supports an endogenous origin of MSRV [116,122]. 

#### 7.1.2. Presence of HERV-W/MSRV Ags in MS Lesions

As is the case for HERV-W/MSRV nucleic acids, the presence of HERV-W/MSRV proteins has been reported in both normal and MS brain tissues, thus questioning their direct role in MS pathogenesis. However, the presence of HERV-W/MSRV proteins in diseased sites suggests that they may contribute to MS immunopathogenicity and clinical manifestations. In fact, Syncytin-1 protein was present in MS patient brains and in specific cell types involved in lesions, neuroinflammation, and were expressed at a low level [118] or absent [107,120] in controls. In addition, Syncytin-1 in vitro expression mediated the production of proinflammatory molecules, potentially involved in astrocytes and oligodendrocyte damage [95], and an accumulation of HERV-W Gag Ags was shown in MS demyelinated brain lesions [123]. Also, HERV-W Env epitopes were detected in higher quantities on the surface of B cells and monocytes from patients with active MS with respect to stable MS patients and healthy controls [124]. Finally, HERV-W/MSRV Env protein abundance in MS brain lesions was recently associated with areas of active demyelination, being predominantly expressed by macrophages and microglia, while moderate expression was observed in reactive astrocytes within active lesions [125].

#### 7.1.3. Onset of an Immune Response against These Elements

Growing evidence suggests that HERV-W/MSRV Env may act as super-Ag that triggers an abnormal innate immune response independently of a specific recognition pathway. This immune activation could lead to the overproduction of cytokines, which are known to play a major role in MS inflammatory demyelinating process. MSRV Env induced in both healthy donors and MS patients the in vitro polyclonal activation of Vβ16 T-lymphocytes [126] and the subsequent increase of multiple pro-inflammatory cytokines [126,127]. These HERV-W/MSRV Env pro-inflammatory properties have been attributed to the protein’s ability to trigger the Toll-Like Receptor 4 (TLR4) [126,128,129], leading to the overexpression of the same proinflammatory cytokines involved in MS, such as interleukins 1 and 6 (IL-1, IL-6) and Tumor Necrosis Factor α (TNF-α), and inducing lymphocyte Th-1 polarization [127,128,130]. Moreover, HERV-W Env interaction with TLR4 and the consequent upregulation of proinflammatory factors, in particular inducible nitric oxide synthase, led to the formation of nitrotyrosine groups, which directly affected myelin expression and remyelination by blocking oligodendrocyte precursor differentiation [131]. In support of these findings, HERV-W Env neutralization by monoclonal Ab GNbAC1 reduced such stress reactions and rescuing myelin expression [132], and MSRV Env was recently confirmed to be a potent agonist of human TLR4 in vitro and in vivo [133]. 

However, some reports assessed the development of a specific humoral response against HERV-W/MSRV in MS patients, and found weaker support for its role in MS pathogenesis. Ruprecht et al. reported the presence of Syncytin-1 Abs in only 1 of 50 MS patients and in none in 59 controls, whereas MSRV Gag or Env Abs were not detected [112]. In a follow up study in MS patients that monitored Abs titers against HERV Env proteins, a decrease in anti-HERV-W Env reactivity as a consequence of interferon (IFN)-β therapy was reported but the decrease was not statistically significant [134], as previously observed for circulating Env RNA [135]. Finally, a study assessing the humoral response against selected HERV-W Env peptides showed that two peptides were strongly recognized by MS patients IgG as compared to controls, and a decrease in recognition after six months of IFN-β therapy was also reported [136].

#### 7.1.4. Use of Some Animal Models of MS

The potential link between HERV-W/MSRV immunopathogenic properties and MS has been investigated through mice models, which generally supported the active involvement of these elements in disease development. Intraperitoneal injection of MSRV virions in immunodeficient mice transplanted with human lymphocytes led to the onset of acute neurological symptoms, causing death by massive brain hemorrhage [137]. Further analysis confirmed the presence of circulating MSRV RNA and splenic overexpression of proinflammatory cytokines [137]. In another study, Syncytin-1 induced neuroinflammation, neurobehavioral abnormalities and oligodendrocyte and myelin injury principally evoked by redox reactant–mediated cellular brain damage [118,138]. Always in mice, MSRV-Env was able to activate the TLR4- and CD14-mediated release of proinflammatory cytokines and, when associated to the myelin oligodendrocyte glycoprotein (MOG) Ag, induced a specific T cells IFN-C production. Such combined innate and acquired responses promoted the development of experimental allergic encephalomyelitis, and was proposed as a suitable MS animal model [139].

In addition to the high number of studies that all assessed the whole group general expression in MS, a limited number of studies were dedicated to the investigation of individual transcribed HERV-W loci. The HERV-W processed pseudogene in locus Xq22.3 (ERVWE2) was among the most highly investigated due to the presence of an almost complete *env* ORF, interrupted only by a premature stop at codon 39. Noteworthy is that this L1-retrotransposed HERV-W element is transcribed in human PBMCs [13,140,141], producing ex vivo an N-terminally truncated Env protein (N-Trenv) [142]. In addition, the evidence that a monoclonal Ab previously used to detect HERV-W Env in MS lesions (13H5A5) [123] was able to bind N-Trenv, but not Syncytin-1, allowed speculation that this and other expressed defective proteins may exert some effects in vivo [142]. Also, the ERVWE2 locus in chromosome X has been proposed as the hypothetical genomic origin of MSRV Env proteins [108,110] and investigated for its potential role in MS and its higher incidence in women. However, analysis of ERVWE2 DNA sequences in MS patients and healthy individuals PBMC revealed that all harbored the stop codon at site 39, and assessed whether genetic polymorphisms could possibly allow the production of a full-length protein in vivo [143]. The authors also identified 5 ERVWE2 DNA regions similar to the MOG Ig-like domain that, together with other co-factors, could trigger the immune cross-reaction against myelin in MS [143]. García-Montojo et al. genotyped the ERVWE2 insertion in a wide group of individuals, and reported a significant association with female MS susceptibility and polymorphisms rs6622139 and rs1290413, which are more frequent in controls than MS affected women [144]. A similar analysis was performed for an HERV-W insertion in chromosome 20, but the two identified polymorphisms were not significantly linked to MS susceptibility based on case–control studies [145]. Other work addressed HERV-W loci expression in MS. Laufer at al. tried to clarify the origin of the HERV-W/MSRV *env* sequences detected in MS samples by evaluating expression from single HERV-W loci. Interestingly, expressed HERV sequences was shown to be often complicated by in vitro recombination between HERV transcripts, probably caused by RT template switches and/or PCR-mediated recombination [13,141]. In particular, the authors proposed that some previously published MSRV *env* sequences, as well as a high number of HERV-W *env* cDNA clones, had actually arisen from the recombination of different HERV-W *env* transcripts, detecting up to four recombination events involving up to five HERV-W loci for the same sequence. It was also shown that the primers commonly used for HERV-W expression studies were similar enough to anneal with multiple HERV-W loci, underlining the importance of precisely assessing the transcripts genomic origin when studying HERV RNA expression [83,141]. Of note, similar individual HERV-W *env* loci expression levels were found in PBMCs from MS patients and healthy controls [141], further supporting the low specificity of RNA transcripts for MS disease. Another comprehensive analysis of HERV-W loci brain transcription was performed by high-throughput sequencing of *env*-specific RT-PCR products, identifying >100 HERV-W loci transcribed at very similar levels in MS patients and healthy individuals [113]. Interestingly, while the deregulated expression of HERV-W *env* in MS brain lesions was refuted, the authors reported an inter-individual variability in HERV-W transcript levels, and a residual promoter activity for many HERV-W LTRs, even if incomplete [113]. A third study analyzing age- and disease-dependent HERV-W *env* RNA diversity showed that HERV-W *env* transcripts originated from multiple loci in primary human neurons, while astrocytes and microglia showed lower diversity in HERV-W transcript chromosomal origin [146]. Similarly, while multiple loci encoding HERV-W *env* RNA sequences were detected in both fetal and adult healthy brains, transcripts cloned from neurologic patients mostly mapped to Syncytin-1 locus (7q21.2), and their abundance was highly correlated with pro-inflammatory gene expression in diseased brains [146]. This could indicate a wide and complex scenario, poorly clarified by the mere upregulation of general HERV-W group expression.

Taken together, HERV-W/MSRV expression analyses in MS patients do not definitively confirm a specific association of these retroviral elements to MS etiology, but strongly suggest a possible role of group expression, especially at the protein level, in disease immunopathogenesis. Variable HERV-W/MSRV expression found in both MS patients and healthy individuals could probably constitute a normal physiological phenomenon, possibly with higher prevalence in MS due to an altered epigenetic and immunological environment [94,147]. This hypothesis opens the possibility of a co-contributing role or predisposing factors that will require additional studies on the HERV-W/MSRV brain proteomic profile in different ethnic populations [147]. In fact, considering the non-specific super-Ag activity of HERV-W/MSRV Env showed neuropathogenic effects coincident with the major hallmarks of MS inflammation [125], the HERV-W group could participate in a complex inflammatory interplay with other not fully understood factors, including genetic predisposition and exogenous infections [97,148]. It is noteworthy that a therapeutic treatment targeting HERV-W/MSRV has been proposed as a possible innovative approach for MS using the GNbAC1 monoclonal Ab developed to selectively recognize MSRV Env. It showed neutralizing effects in vitro and in MS mouse models and is currently in phase II clinical development [149,150].

### 7.2. Other Autoimmune Diseases

Besides MS, a few studies investigated HERV-W group expression in other disorders with poorly understood etiology, in which autoimmunity mechanisms play a major pathogenic role, such as rheumatoid arthritis (RA), osteoarthritis (OA), chronic inflammatory demyelinating polyradiculoneuropathy (CIDP), psoriasis and lichen planus (LP). In all these disorders, no evidence of an etiological link between HERV-W expression and pathogenesis has been reached yet, mostly due to the poor sample representation and failure to assign the observed transcripts to individual HERV-W loci.

RA is characterized by the progressive destruction of articular components and leads to severe disability. A common sign of the RA autoimmune response is the presence of a cellular infiltrate of neutrophils, lymphocytes, and macrophages in the synovial tissue, accompanied by the increased production of metalloproteinases contributing to extracellular matrix erosion [151]. Based on preliminary results reporting HERV-W/MSRV RNA in the 50% of RA patient plasma samples, Gaudin et al. determined if particle-associated HERV-W/MSRV RNA were present in patient samples [151]. The results showed that neither the patients nor the controls had HERV-W/MSRV RNA in plasma, while such RNA was detected in the synovial fluid samples of two out of nine RA patients and one control, suggesting its lack of specificity with respect to RA etiology [151]. 

OA is another common form of arthritis characterized by the progressive destruction of articular cartilage, in which many factors, including viral infections, seem to play a role [152]. Bendiksen et al. analyzed cartilage and chondrocytes from advanced OA as compared to early/non-OA individuals. While all samples were negative for a number of exogenous infections, a HERV-W *env* gene was commonly expressed in advanced OA patient cartilage (88% of patients) while expression was detected in a lower proportion of controls (0–38%) [152]. The authors also reported the abundant expression of Env proteins in OA-derived chondrocytes, and the occurrence of viral budding and virus-like particles. However, the particles were neither isolated nor characterized [152]. 

Another pathology tentatively linked to HERV-W/MSRV is CIDP, a rare immune disease of the peripheral nervous system characterized by inflammatory and demyelinating lesions in nerve roots [153]. Driven by the presence of MSRV-Env in a small number of CIDP patients (5 out of 8) [120], Faucard et al. confirmed an upregulation of MSRV *env* and/or *pol* mRNAs in ~50–65% of CIDP patients PBMC [153]. The authors also reported the presence of MSRV Env protein in 5 out of 7 CIDP patients nerve lesions and dominant expression in Schwan cells [153]. Moreover, Schwan cell cultures exposed to MSRV-Env displayed a potent induction of IL-6 and CXCL10 chemokines, which could be significantly inhibited by GNbAC1 MSRV-Env mAb [153]. 

Finally, HERV-W/MSRV expression may contribute to some skin diseases with unclear etiology. Psoriasis is a chronic disease characterized by epidermal proliferation and abnormal keratinocytes differentiation and shows similarities to systemic immunological disorders closely related to autoimmunity [154]. Considering that HERV expression has been reported in human skin, being either activated or repressed by UV irradiation [155,156], Molès et al. assessed HERV expression in psoriatic lesions. Their work detected various *pol* amplicons comprising HERV-W sequences in both psoriatic and control skin samples [154]. Another pathology taken into account was LP, a chronic skin inflammatory disease characterized by lichenoid papules and possibly involving also microbial agents in its unclear etiology [157]. de Sousa Nogueira et al. observed a downregulation of some HERV groups, including HERV-W *env*, in skin biopsies of LP patients, with a concomitant activation of antiviral restriction genes APOBEC3G, MxA, and IFN-inducible genes that may be involved in immune control of HERV transcription [157].

## 8. HERV-W Expression in Neurological and Neuropsychiatric Disorders

HERV-W neuropathogenic effects have also been investigated in a number of neurological and neuropsychiatric diseases with poorly understood etiology, such as Motor Neuron Disease (MND), sporadic Creutzfeldt–Jakob disease (sCJD), autistic spectrum disorder (ASD), attention deficit hyperactivity disorder (ADHD), and schizophrenia. In general, the available information does not yet support a direct role of HERV-W group in any neurological or neuropsychiatric diseases. In fact, a proportion of HERV-W-negative patients is reported in the majority of the studies, while a significant upregulation of HERV-W expression was shown in a subset of cases, strongly suggesting the presence of other major factors contributing to a complex and poorly understood etiology. It is also worth noting that many of these pathologies could be concomitant with behavioral variables, such as drug and alcohol abuse [158], which could be confounding if they are able to influence HERV brain expression [159]. 

MND is a heterogeneous group of neurologic disorders characterized by the progressive degeneration of motor neurons. Elevated levels of HERV-W *env* transcripts were observed in biopsies from MND patients limbs as compared to control tissues from the same individual and from healthy donors [160]. The authors also detected a parallel upregulation of the SOD1 (oxidative stress-responsive) gene, a marker for oxidative stress, suggesting that its activation could be due to the primary loss of motor neurons instead of being a direct consequence of HERV-W Env neurotoxic effects [160]. 

sCJD is a rare form of prion disease, causing fatal neurodegeneration and having as key event the conformational change of cellular prion protein to an abnormal protease-resistant isoform. Joang et al. examined the expression of 10 HERV groups in sCJD patients CSFs, detecting transcripts of all analyzed groups and reporting the highest incidence for HERV-W *pol* (82.5% positivity), with a significant increase with respect to controls [161]. Based on subsequent subcloning analysis, all observed transcripts showed non-identical nucleotide sequence, and none had specificity for sCJD [161]. 

ASD and ADHD are two neurodevelopmental diseases caused by complex interactions with not fully clarified genetic and environmental factors. ASD patients PBMC showed higher positivity for HERV-H and HERV-W mRNAs as compared to controls [162]. Subsequent quantification showed that HERV-H and HERV-W were, respectively, more and less abundantly expressed in ADS patients [162]. Similarly, the HERV-H transcript level in ADHD patients PBMC was significantly higher, while no differences were found in HERV-W expression [163]. 

Among neuropsychiatric disorders, the field of greatest interest for HERV-W involvement is schizophrenia. The first findings were provided by Deb-Rinker et al. in monozygotic twins discordant for schizophrenia, presenting one sequence (schizophrenia associated retrovirus, SZRV-1, AF135487) similar to both a MSRV (AF009668) and a HERV-9 (S77575) sequences expressed in placenta [164]. Karlsson et al. then detected HERV-W/MSRV *pol* sequences in the cell-free CSF from ~29% acute onset schizophrenia patients and 5% individuals in later stages of the disease, but not in patients with non-inflammatory neurological diseases and healthy controls [165]. Similarly, HERV-W/MSRV expression was up-regulated in the brain frontal cortex regions of schizophrenia patients when compared with control tissues from healthy individuals [165]. In subsequent studies, the same authors reported the presence of HERV-W RNA in the plasma of a subgroup (9 out of 54) of recent-onset schizophrenia patients, 5 of which harbored HERV-W/MSRV sequences in CSF [166]. They detected an elevated level of HERV-W *gag* (but not *env*) transcripts in PBMC of patients with schizophrenia-related psychosis, and reported an upregulation of HERV-W sequences from locus 11q13.5 [167]. HERV-W *env* plasmatic mRNA was found in 36% of recent-onset schizophrenia patients and in none of the 106 controls, and also RT activity was significantly increased in patient sera [168]. At the protein level, HERV-W Env hyperexpression in U251 human glioma cells triggered the production of the dopamine receptor D3 and brain-derived neurotrophic factor (BDNF), both associated with schizophrenia, and increased the phosphorylation of CREB protein, which is necessary for BDNF expression [168] and confirmed in human neuroblastoma cells [79]. Moreover, recent findings also suggested that phosphorylation of Glycogen Synthase Kinase 3β might be involved in HERV-W Env-mediated BDNF induction [169]. A study detecting HERV-W Ags in patients reported positive serum antigenemia for Gag and Env in ~50% of schizophrenic patients and in 3–4% of blood donors [170]. Of note, a full-length HERV-W LTR was found in the regulatory region of GABBR1 (GABA receptor B1) gene, which is downregulated in schizophrenic patients [171]. However, the roles of this LTR and GABBR1 in schizophrenia remain to be clarified.

In contrast to the studies reported above, which showed an increased HERV-W expression in Schizophrenia [166,167,168], a number of investigations reported no specific correlation between the HERV-W transcription and development of neurological diseases [158,172,173]. The comprehensive microarray-based analysis of 20 HERV group’s transcriptional activity in 215 brain samples from schizophrenia or bipolar disorders (BD) patients and matched controls failed to show relevant links between HERV brain expression and schizophrenia, suggesting that it could be more likely that HERV transcriptional activity is influenced by the individual genetic background and the presence of immune cell infiltrates and/or medical treatments [172]. Interestingly, the different brain areas of each individual showed a common pattern of HERV expression, where the HERV-W *env* gene was transcriptionally active but did not show significant differences between healthy controls and schizophrenic patients [172]. Weis et al. observed that HERV-W Gag proteins were present in human brain anterior cingulate cortex and hippocampus, mostly associated with neurons and astrocytes, and showed significantly reduced expression in schizophrenia, major depression, and BD patients as compared to controls [158]. HERV-W *env* transcription was increased in schizophrenia and BD patient PBMCs, but the corresponding DNA copy number was paradoxically lower in patients than in healthy controls. Moreover, differences in HERV-W *env* amplicon nucleotide sequences and their relative frequencies were observed in comparisons of patients to controls and in comparisons among Schizophrenia and BD patients to MS patients [173]. The authors hypothesized that when HERV-W genes are hypomethylated during development, environmental stimuli (such as exogenous infections) could prompt lineage-specific HERV-W genomic modifications and determine variable patterns responding differently to subsequent environmental triggers, leading to diverse clinical manifestations [173]. 

## 9. HERV-W Expression in the Presence of Exogenous Infections

HERVs have also been proposed to influence exogenous viral infections, and such a role could be either beneficial or harmful. HERV antisense transcripts have been hypothesized as a plausible defense against exogenous infections, in which complementarity between homologous retroviral RNA sequences could form dsRNA and detected as a PAMP by the innate immunity PRRs [89,174]. Another possible HERV-mediated antiviral effect could be the partial resistance to infection, evoked by receptor interference and blocking by HERV proteins [175,176]. However, exogenous viruses and expressed HERVs may also generate cooperative effects, stimulating each other’s transcription or leading to the complementation of defective elements. Clearly, some of these interactions require a certain degree of sequence and structural homology, and are most likely to happen between HERVs and exogenous retroviruses.

### 9.1. Retroviral Infections

Humans are currently threatened by two exogenous retroviruses: Human Immunodeficiency Virus (HIV, *Lentiviridae*) and Human T-cell Lymphotropic Virus (HTLV, *Deltaretroviridae*). HERV-W Env glycoprotein was shown to functionally complement an *env*-defective HIV-1 strain, generating HERV-W-pseudotyped particles infectious for CD4-negative cells, and therefore, possibly expanding HIV-1 tropism [177]. HERV-W elements were upregulated in three persistently HIV-1 infected cell lines, but not in infected cells [178]. Of note, reversal of HIV-1 latency by treatment with histone deacetylase inhibitors caused no substantial increases of HERV-W *env* gene transcription [179]. Significant HERV-W RNA hyperexpression was detected in the brains of AIDS patients suffering from dementia [114]. However, this variation in expression is probably a consequence of increased immune activity linked to monocyte differentiation and macrophage activation, and had no active role in AIDS neuropathy. HIV Tat transactivator protein increased MSRV *env* mRNAs and HERV-W Env protein expression in astrocytes and differentiated macrophages but reduced expression in monocytes [180]. Similarly, HTLV-I Tax homolog of Tat also activates transcription from HERV-W LTRs by interacting with CREB along with other transcription factors [181]. T cell cross-reactivity between HERV and HIV epitopes was tested in vitro, giving negative results [182].

### 9.2. Herpesviral Infections

The possible interplay between Herpesviruses infection and HERV-W expression has been widely analyzed, especially in the context of MS and autoimmunity, and the ability of various Herpesviruses to influence HERV-W transcription [85,86,87]. HERV-W/MSRV expression was enhanced by Herpes Simplex Virus 1 (HSV-1) superinfection in MS patients cells [41,96,183]. More specifically, Lafon et al. showed that HERV-W Env protein expression in neuroblastoma cell lines can be reactivated by HSV-1, probably through its infected cell polypeptide 0 and 4 (ICP0 and ICP4, respectively) early proteins [96]. HERV-W Gag and Env proteins were also induced by HSV-1 in neuronal and brain endothelial cells in vitro, and expression was also compatible with an ICP0-mediated activation [184]. Additional evidence has been reported in HeLa cells, in which HSV-1 IE1 protein stimulated LTR-driven transcription of HERV-W elements, probably through the modulation of the Oct-1 transcription factor [185]. The authors proposed that IE1 activation could also involve HERV-W solitary LTRs, potentially promoting possible nearby genes [185].

Besides HSV-1, other Herpesviruses have been analyzed for their ability to activate HERV-W in MS. A hypothetical ERVWE1 Env peptide (29 aa) harbored an epitope predicted to be presented by different HLA class I molecules and possibly acted as a target for effector T-cells in MS. Interestingly, this epitope was partially homologous with all the pathogens against with elevated Abs titers in MS patients, including HSV-1, HHV-6, VZV (Varicella Zoster Virus), EBV (Epstein Barr Virus), and measles virus [186]. Hence, it was claimed that the effector T cell recognizing this putative epitope would most readily cooperate with regulatory T cells to support an immune response, leading either to a prompt resolution of the infection or to tissue damage by autoimmune processes [99,186]. Regarding EBV, the exposure to the virus or to its major Env glycoprotein (gp350) triggered HERV-W/MSRV expression in PBMCs from MS patients and MSRV positive healthy controls, as well as in cultured U87-MG astrocytes, with an activation pathway possibly involving NF-kB [187]. The infection of a number of cancer and non-cancer cell lines with CMV induced RT activity in all cells, and upregulated various HERVs, including HERV-W, in CMV-infected neural tumor stem cells after UV irradiation [188]. Other evidence of a helper role in HERV-W activation came from kidney transplant recipients with high CMV load, who displayed significantly higher HERV-W *pol* expression than patients with moderate/undetectable CMV load or healthy subjects [189]. 

### 9.3. Other Exogenous Infections

Although retroviral and herpesviral infections have been most intensively studied for their effects on HERV-W expression, influenza virus, spleen necrosis virus (SNV) and porcine endogenous retrovirus (PERV) infections have also been implicated in modulating HERV-W.

Nellåker et al. described specific expression patterns of HERV-W *gag* and *env* genes (even if encoded by sequences with truncated/no LTRs) in different cell-lines, and observing subsets of elements being transactivated by influenza virus active replication [140]. Similar variations were also observed as a consequence of serum deprivation, suggesting that the cellular stress itself could contribute to HERV-W modulation [140]. Subsequent analysis showed that influenza virus infection induced spliced ERVWE1 transcripts able to encode Syncytin-1 in extra-placental cells by GCM1 overexpression [190], and downregulated the level of repressive histone mark H3K9me3 [191].

The HERV-W Env glycoprotein induced cellular resistance to SNV, whose infectivity was reduced by 1000-10,000-fold in D-17 cells expressing HERV-W Env [192].

Finally, in the field of xenotransplantation, the expression level of HERV-W genes differed in PERV-infected HEK-293 cells in comparison to uninfected cultures [193].

## 10. The HERV-W Transcriptional Landscape in the Context of Human Physiopathology: Current Needs and Future Perspectives

As described in the previous sections, no definitive link between HERV-W expression and disease onset and progression has been found. The great majority of the studies, in fact, have been based on the detection and the eventual quantification of expression from the entire HERV group. The analysis of bulk HERV expression cannot reveal the contribution of specific HERV-W loci to physiological or pathological disease states. Furthermore, most of the studies have been based on not fully standardized methodologies performed on differently representative samples. Thus, although a great amount of data has been generated, they are frequently discordant and difficult to compare with each other. The main biases that affected, in our opinion, a great number of studies aimed at analyzing the HERV-W group expression are summarized in Table S2, along with their inconclusive nature and suggestions for improvement. 

The currently available information strongly suggests that the HERV-W group is commonly transcribed in human cells, and that this happens in both healthy and pathological conditions. Collective expression greatly varies between tissues and reflects individual genetic backgrounds. In particular, the presence of HERV-W RNA and Ags in human tissues and cells seems to be a physiological phenomenon. HERV-W expression is detected in (a subset of) healthy individuals and possibly increases under pathological conditions, without necessarily representing an etiological factor. The direct pathogenic effects of HERV-W RNA and proteins have not been confirmed and still lack a molecular mode of action in vivo. In contrast, the effects that these HERV-W products could trigger in their interplay with the host—including immune stimulation, insertional mutagenesis (also through de novo retrotransposition events) and deregulatory functions—are more likely to contribute to human pathogenesis, and also influence some animal models of human disease. Even in this case, however, the identification and characterization of the specific HERV-W sequences and the exact molecular mechanisms involved remains a major goal and necessary for the exploitation of HERV-W candidates for therapeutic purposes.

Defining the expression patterns of single HERV-W loci through a dedicated microarray or RNA-seq analyses will be essential to provide additional insights into the quantitative changes originating from specific HERV-W sequences and to identify single members possibly linked to human pathogenesis. Indeed, this promising field deserves a deeper investigation aimed, first of all, at characterizing all the possible mechanisms involving HERV-W presence and expression in both physiological and pathological conditions. The latter also includes the possibility that an altered epigenetic environment could prompt de novo mobilization of HERV-W transcripts by active L1 elements. This ongoing process would generate additional HERV-W processed pseudogenes when compared with the ones recently mapped in the human genome reference sequence [21].

The current lack of knowledge of the individual HERV-W loci transcriptional status is in large part due to the previous incomplete characterization of the HERV-W genomic landscape. Much of the current experimental designs have focused on a few HERV-W sequences, above all Syncytin-1 and MSRV clones (Table S2). This has prevented until now (i) the univocal assignment of the HERV-W expressed sequences to the locus of origin; (ii) the characterization of the single HERV-W sequences differential expression in the diverse physiological tissues, essential to assess their effective dysregulation in diseased environments; (iii) the evaluation of the full-length HERV-W sequence transcription, coding capacity and regulatory elements; (iv) the characterization of the single HERV-W sequences epigenetic status in both physiological and pathological contexts; and (v) the study of the HERV-W sequences genomic context of insertion, identification of nearby host genes that can potentially influence (or be influenced by) HERV-W elements even in the absence of a detectable expressed product (Table S2).

We also outline future research investigating HERV-W’s contribution to human physiopathology, including dedicated genome-wide high-throughput studies using stringent primers and probes that are able to distinguish the uniqueness of single HERV-W elements conforming to standard conditions. Developing these reagents will help properly define the specific contribution of the different retroelements to the human transcriptome [95]. Importantly, the physiological consequences of individual HERV-W loci expression must be evaluated a priori, in order to have a reliable “basal” level to compare with the same, diseased, tissue [86]. Such specific quantitative analyses must then be performed on a statistically significant population, possibly including paired samples of both healthy tissues and pathological lesions from the same individual. Moreover, these investigations also take into account the influence of the HERV-W loci genomic context of integration, and to analyze the molecular diversity of single insertions within the human population. HERV-W polymorphisms are necessary to understand due to the possibility that different HERV-W allelic variants may exert specific effects on the pathogenesis phenotype, progression and therapeutic response, depending on the host genetic background [95]. Considering that ~80–100 copies of L1 are active in the human genome [36,39,194], the eventual L1-mediated retrotransposition of HERV-W sequences should also be considered, especially in those pathological environments associated with an altered epigenetic control, where the hypomethylation of both L1 and HERV-W sequences have been reported [74]. Finally, since structurally-incomplete LTRs could be still able to drive the transcription of HERV-W proviruses, processed pseudogenes or nearby host genes, the methylation levels of truncated and solitary LTRs should also be evaluated. Indeed, besides detecting HERV-W group overall expression, the identification of the specific encoding locus appears to be mandatory to establish any definitive associations between human diseases and specific retroelements, and also to properly understand the molecular nature of emergent forms that have arisen through recombination events involving different HERV-W loci [95], especially in those contexts where the epigenetics alteration could liberate HERVs expression. Similarly, the molecular determinants responsible for specific HERV-W loci upregulation as well as their role either as a cause or a consequence of disease must also be clarified in detail [97]. Together, these approaches will finally provide well-characterized mechanisms of HERV-mediated pathogenesis.

In conclusion, it is certainly possible and perhaps likely that specific HERV-W sequences may play a role in human pathogenesis, without necessarily being the only etiological determinant of disease. Indeed, in the field of autoimmunity, one or more HERV-W insertions (or even specific allelic variants) and/or their expressed products may be involved in a complex inflammatory and immune interplay with other unknown or not fully understood co-factors. The latter may include individual predispositions, depending on the host genetic background, as well as extrinsic factors such as stress, environmental stimuli or exogenous infections. All these complex relationships must be considered, especially in the field of multifactorial disorders with poorly clarified etiology. In light of this, the identification and characterization of the precise HERV-W loci showing a differential transcription pattern and/or L1-mediated HERV-W de novo mobilization in a specific pathological context appear to be mandatory to definitively demonstrate a cause–effect connection to any disease etiology, and to subsequently identify single HERV-W sequences exploitable as novel therapeutic targets. The latter could be suitable for various, innovative approaches, from the employment of retroviral inhibitors to the administration of passive as well as active immunotherapy directed against specific HERV products, possibly in association with the treatment with DNA-demethylating agents. However, even in the absence of an etiological contribution, the identification of specific HERV-W sequences selectively expressed in a given pathological context could provide novel and reliable sequence-based biomarkers of disease. Also, disease-associated Ags suitable for directing immunotherapeutic approaches to the precise site of pathogenesis could be developed as protein biomarkers. All these HERV-based therapeutic applications could certainly constitute an innovative treatment for many human diseases.

## Figures and Tables

**Table 1:** General type W human endogenous retroviruses (HERV-W) group expression in non-placental healthy tissues.

Tissue	Method	Ref.	Possible Biases ^a^
Blood	Search of Syncytin query in EST data	[11]	Low total HERV EST counts, could not detect HERV-Ws divergent from Syncytin, no information on LTR activity, number of cDNA/EST libraries great variability across tissues, under-representation of poorly expressed genes in small libraries (1)
Brain	Search of Syncytin query in EST data	[11]	(1)
	RT-PCR (*gag+*, *pol+*, *env+*)	[55]	Primers specific for single expressed sequences (placental Syncytin (*gag:* AF072500, *env:* AF072506), MSRV clones (*pol:* AF009668)) could not detect divergent HERV-Ws, no information on full-length HERVs expression and LTR activity, samples amount is poorly representative (2)
Brain (cortex and pons)	*env* real time qRT-PCR	[56]	Primers specific for placental Syncytin (NM_014590.3) can could not detect *env* defective or highly divergent HERV-Ws, no information on full-length HERVs expression and LTR activity, samples amount is poorly representative (3)
Breast	Search of Syncytin query in EST data	[11]	(1)
	*env* real time qRT-PCR	[56]	(3)
Colon	*env* real time qRT-PCR	[56]	(3)
Heart	RT-PCR (*gag−*, *pol−*, *env+*)	[55]	(2)
Endometrium	GammaHERV and HERV-W *pol*-based probe and probe-less real time qPCRs	[57] [14]	Could not detect transcripts defective or highly divergent for *pol* gene, no information about full-length sequences expression and LTR activity, samples amount is poorly representative (4)
Kidney	*pol*-expression arrays hybridization	[10]	Cross-amplification/hybridization of related HERV groups; could not detect transcripts defective for *pol* gene, no information about full-length sequences expression and LTR activity, no quantitative information, samples amount is poorly representative (5)
	RT-PCR (*gag−*, *pol+*, *env+*)	[55]	(2)
Liver	*pol*-expression arrays hybridization	[10]	(5)
	RT-PCR (*gag−*, *pol+*, *env+*)	[55]	(2)
	*env* real time qRT-PCR	[56]	(3)
Liver-spleen (fetal)	Search of Syncytin query in EST data	[11]	(1)
Lung	RT-PCR (*gag−*, *pol+*, *env+*)	[55]	(2)
Ovary	Search of Syncytin query in EST data	[11]	(1)
	GammaHERV and HERV-W *pol*-based probe and probe-less real time qPCRs	[57] [14]	(4)
PBMC	*pol* RT-PCR and *env* real time PCR	[17]	Low sensitivity and cross-amplification of related HERV groups by RT-PCR degenerate primers, real time PCR primers specific for placental Syncytin (NM_014590.3) could not detect divergent HERV-Ws and transcripts defective for *pol/env* genes, no information on full-length sequences expression and LTR activity, incomplete characterization of individuals health status
Prostate	RT-PCR (*gag−*, *pol+*, *env+*)	[55]	(2)
Skel. Muscle	RT-PCR (*gag−*, *pol+*, *env+*)	[55]	(2)
Spleen	RT-PCR (*gag+*, *pol+*, *env+*)	[55]	(2)
Stomach	*env* real time qRT-PCR	[56]	(3)
Testis	RT-PCR (*gag+*, *pol+*, *env+*)	[55]	(2)
Thymus	RT-PCR (*gag−*, *pol+*, *env+*)	[55]	(2)
Uterus	RT-PCR (*gag−*, *pol−, env+*)	[55]	(2)
	*env* real time qRT-PCR	[56]	(3)

General HERV-W expression was reported by Stauffer et al. in the blood, brain, breast, liver/spleen, ovary and placenta, and subsequent analysis confirmed such results for placental and breast tissues only [11]. The physiological HERV-W *env* transcription in healthy brain and breast was detected also by Kim et al. [56]. Yi et al. investigated the HERV-W *gag*, *pol* and *env* genes expression within 12 tissues (brain, prostate, testis, heart, kidney, liver, lung, placenta, skeletal muscle, spleen, thymus, and uterus), detecting *env* transcripts in all the analyzed samples and reporting also some tissue-specific expression for *gag* and *pol* [55]. HERV-W RNA expression was also reported in the normal endometrium and ovary [14,57] and in the colon, liver, stomach, and uterus [56]. The HERV-W group was found to be transcriptionally active in peripheral blood mononuclear cells (PBMC) since early childhood [17]. High resolution melting temperature analysis [58] assessed the occurrence of systematic variations in the HERV-W *gag* sequences expression in primary fibroblasts, depending on both tissues and individuals considered [59]. ^a^ Methodological biases potentially affecting the effective and specific detection and characterization of the expressed HERV-W sequences. After the first citation, biases with multiple citations are reported as a number into round brackets. EST: expressed sequence tags; LTR: long terminal repeats; MSRV: multiple sclerosis retrovirus; qRT-PCR: quantitative reverse transcriptase PCR; PBMC: peripheral blood mononuclear cells.

**Table 2:** Specific HERV-W loci for which an expression in healthy tissues has been reported.

Locus	Chr:start-end (Strand) ^a^	Type	Genomic Context ^b^	Tissue	Method	Ref.
2q22.1	2:139030735-139031481 (−)	Solo LTR	*LTR8* (+)	Testis	Microarray	[15]
2q24.3	2:165514421-165516121 (−)	Pseudogene	COBLL1 (−) *TCONS_00004484* (−)	Placenta	Microarray	[15]
5q12.1 *	5:59954322-59962280 (+)	Provirus	DEPDC1B (−)	Placenta	Microarray	[15]
7q21.2 *	7:92097313:92107506 (−)	Provirus	-	Placenta Testis	Northern blot	[23] [25]
15q21.2	15:51552784-51553570 (+)	Solo LTR	CYP19A1 (−)	Placenta	Microarray	[15]
Xq21.33	X:93824238-93824702 (−)	Solo LTR	MER4A (−)	Placenta	Microarray	[15]

^a^ Chromosomal positions are referred to genome assembly GRCh37/hg19. The Syncytin locus is highlighted in bold. ^b^ Localization of HERV-W element within a human gene (italic names correspond to non-coding elements). For sequences marked with an * LTR promoter activity has been also reported.

**Table 3:** General HERV-W group expression in tumoral tissues.

Tumoral Tissue	Ref.	Method ^a^	Physiol. Expression ^b^	Possible Biases of HERV-W Members Underrepresentation ^c^
B cells	[55] *	RT-PCR (*gag−*, *pol−*, *env+*)	[17] °	Primers specific for single expressed sequences (placental Syncytin-1 *gag* AF072500 and *env* AF072506; MSRV clones *pol* AF009668) could not detect divergent HERV-Ws, no information on full-length HERVs expression and LTR activity, samples amount is poorly representative (2)
Bladder	[55] *	RT-PCR (*gag−*, *pol+*, *env+*)	-	(2)
Breast	[11]	Search of Syncytin-1 in EST data	[11,56]	Low total HERV EST counts, could not detect HERV-Ws divergent from Syncytin-1, no information on LTR activity, number of cDNA/EST libraries great variability across tissues, under-representation of poorly expressed genes in small libraries (1)
	[76] *	RT-PCR, real time qRT-PCR,		Specific detection of a Syncytin-1 *env* portion only, could not detect transcripts divergent/defective for *env*, no information on full-length sequences expression and LTR activity
	[56]	*env* real time qRT-PCR		Primers specific for placental Syncytin-1 (NM_014590.3) could not detect *env* defective or highly divergent HERV-Ws, no information on full-length HERVs expression and LTR activity, samples amount is poorly representative (3)
	[55] *	RT-PCR (*gag−*, *pol+*, *env+*)		(2)
Brain	[55] *	RT-PCR (*gag−*, *pol+*, *env+*)	[11,55]	(2)
Colon	[11]	Search of Syncytin-1 in EST data	[56]	(1)
	[56]	*env* real time qRT-PCR		(3)
	[55] *	RT-PCR (*gag−*, *pol+*, *env+*)		(2)
	[77] *	qPCR		Specific detection of a Syncytin-1 *env* portion only, could not detect transcripts divergent/defective for *env*, no information on full-length sequences expression and LTR activity
Endometrium	[75]	qPCR, RT-PCR, NB, WB	[14,57,75]	Specific detection of a small portion of Syncytin-1 *env* only, samples amount is poorly representative, expression values are highly heterogeneous
Esophagus	[55] *	RT-PCR (*gag−*, *pol+*, *env+*)	-	(2)
Histiocyte	[55] *	RT-PCR (*gag−*, *pol+*, *env+*)	-	(2)
Kidney	[11]	Search of Syncytin-1 in EST data	[10,55]	(1)
	[55] *	RT-PCR (*gag−*, *pol+*, *env+*)		(2)
Liver	[56]	*env* real time qRT-PCR	[10,55,56]	(3)
	[55] *	RT-PCR (*gag−*, *pol+*, *env+*)		(2)
Lung	[55] *	RT-PCR (*gag−*, *pol+*, *env+*)	[55]	(2)
Neuroblasts	[78] *	*pol* real time qPCRs	-	Could not detect transcripts defective or highly divergent for *pol* gene, no information about full-length sequences expression and LTR activity, samples amount is poorly representative (4)
Ovary	[74]	Real time qRT-PCR	[57,74]	Primers designed on Syncytin-1 locus (AC000064) could not detect divergent HERV-Ws, samples amount is poorly representative
	[57]	*pol* real time qPCRs		(4)
	[55] *	RT-PCR (*gag−*, *pol+*, *env+*)		(2)
Pancreas	[55] *	RT-PCR (*gag−*, *pol+*, *env+*)	-	(2)
Placenta	[11]	Search of Syncytin-1 in EST data	[23,24,25]	(1)
Prostate	[55] *	RT-PCR (*gag−*, *pol−*, *env+*)	[55]	(2)
Skin	[55] *	RT-PCR (*gag−*, *pol−*, *env+*)	-	(2)
Stomach	[56]	*env* real time qRT-PCR	[56]	(3)
	[55] *	RT-PCR (*gag−*, *pol+*, *env+*)		(2)
T-cells	[55] *	RT-PCR (*gag−*, *pol+*, *env+*)	[17] °	(2)
Uterus	[56]	*env* real time qRT-PCR	[55,56]	(3)
	[55] *	RT-PCR (*gag−*, *pol+*, *env+*)		(2)

^a^ NB = Northern Blot, WB = Western Blot; ^b^ Studies that reported the general group expression in healthy tissues; ^c^ Methodological biases that potentially affected the effective and specific detection and characterization of the expressed HERV-W sequences. After the first mention, biases with multiple citations are reported as a number; ° data obtained in total PBMC; * data obtained in cancer cell lines.

**Table 4:** Specific HERV-W loci reported as hyperexpressed in tumoral tissues.

Locus	Chr:start-end (Strand) ^a^	Type ^b^	Genomic Context ^c^	Tissue ^d^	LTR ^e^	Method ^f^	Ref.
1q31.2	1:192855545-192856320 (−)	LTR	MER21C (−)	Testis	-	MA	[15]
2p24.2	2:17520208-17527981 (+)	PV	L3 (−)	Testis	Pro °	MA, qRT-PCR	[15,80]
2p12	2:76098816-76106624 (+)	PV	-	Testis	Pro	MA	[15]
3p12.3	3:74921984-74927237 (−)	PG	-	Prostate	-	MA	[15]
3q28	3:191376573-191383381 (+)	PG	-	Testis	-	MA	[15]
4p13	4:42287455-42294913 (−)	PV	TCONS_00007753 (−)	Testis	Pro °	MA, qRT-PCR	[15,80]
4q26	4:114965536-114972972 (+)	PG	-	Testis	-	MA	[15]
5p13.3	5:31109366-31109859 (−)	LTR	-	Ovary	-	MA	[15]
6q21	6:106676012-106683689 (+)	PG	*ATG5* (−)	Skin T cells	-	MA, qRT-PCR	[81]
7q21.2	7:92097313:92107506 (−)	PV	-	Testis*	Pro °	MA, qRT-PCR	[80]
				Bladder	Pro	qRT-PCR	[82]
				Skin T cells	-	MA, qRT-PCR	[81]
7q21.3	7:95987661-95988433 (−)	LTR	Alu Sx (−)	Testis	-	MA	[15]
7q31.1b	7:114019143-114026368 (−)	PG	*FOXP2* (+)	Testis	-	MA	[15]
7q36.3	7:155177752-155178503 (−)	LTR	BC150495 (+)	Testis	PA	MA	[15]
8q24.13	8:125912007-125919468 (−)	PV	-	Prostate	Pro	MA	[15]
13q21.1	13:55627766-55635877 (+)	PV	-	Testis	-	MA	[15]
13q21.33	13:69795752-69799468 (+)	PV	LINC00383 (+) (Ex)	Testis	Pro °	MA, qRT-PCR	[80]
16p12.3	16:18124951-18125494 (−)	LTR	-	Testis	-	MA	[15]
17q22	17:53088886-53095859 (−)	PG	*STXBP4* (+)	Testis	-	MA	[15]
21q21.1	21:20125060-20132866 (−)	PV	MIR548XHG (−) (Ex)	Testis	-	MA	[15]
21q21.3	21:28226756-28234297 (+)	PV	-	Testis	Pro °	MA, qRT-PCR	[15,80]
Xq21.1	X:82517449-82517774 (−)	LTR	L1 PA11 (+), L1 MA2 (+)	Testis	-	MA	[15]
Xq23	X:113140352-113141135 (−)	LTR	L1 (−), XACT (−)	Testis	Pro °	MA, qRT-PCR	[80]
Xq24	X:120490096-120490859 (+)	LTR	-	Testis	PA	MA	[15]

^a^ Chromosomal positions are referred to genome assembly GRCh37/hg19. Syncytin locus is highlighted in bold; ^b^ PV: provirus; PG: processed pseudogene, LTR: solitary LTR; ^c^ Elements co-localized with HERV-W loci: italics indicates coding genes, (Ex) indicates HERVs within an exon; ^d^ Tissues for which the HERV-W sequence expression was reported also in physiological conditions are marked with *; ^e^ Reported activity of the sequences LTRs: Pro: promoter; PA: PolyA signal. The mark ° indicates a hypomethylated status with respect to normal samples; ^f^ MA: microarray.

## Supplementary Materials

The following are available online at www.mdpi.com/1999-4915/9/5/162/s1. Table S1: Syncytin-1 expression in pathological placentas, Table S2: Major biases and current needs for HERV-W transcriptome studies.
