# Supplementary material for: Type W Human Endogenous Retrovirus (HERV-W) Integrations and Their Mobilization by L1 Machinery: Contribution to the Human Transcriptome and Impact on the Host Physiopathology
Source: Viruses. 2017 Jun 27;9(7):162. doi: 10.3390/v9070162 (PMC5537654; doi:10.3390/v9070162)
Supplement: Supplementary file 1 [file viruses-09-00162-s001.pdf]

Table S1: Syncytin-1 expression in pathological placentas

Pathology	Syncytin-1 expression	Clinical Manifestations	Ref.
Pre-eclampsia	↓ Syncytin-1 expression [a]	↓ trophoblasts fusion *<br>↓ trophoblasts differentiation *<br>↑ hypoxia	[43,64–67]
HELLP syndrome	↓ Syncytin-1 expression	↓ trophoblasts arterial transformation<br>↑ hypoxia	[64,65,68,69]
Trisomy 21	↓ Syncytin-1 expression	↓ trophoblasts fusion *<br>↑ hypoxia<br>↑ trophoblasts apoptosis	[70–73]
IntraUterine Growth Restriction	↓ Syncytin-1 expression	↓ trophoblasts fusion *<br>↑ hypoxia	[66,74]
Endometriosis	↑ Syncytin-1 expression [b]	?	[57,58,75]

[a] Syncytin-1 promoter was hypermethylated

[b] Syncytin-1 promoter was hypomethylated

Clinical manifestations representing a direct effect of Syncytin-1 altered expression are marked with a *.

Table S2. Major biases and current needs for HERV-W transcriptome studies

Common biases	Consequence	Future needs and perspectives
Use of Syncytin-1/MSRV clones/HERV-W clones to design primers and probes and as a query	Lack of detection of transcripts with defective/divergent sequence, detection of recombinant chimeric transcripts	Use of dedicated primers/probes mapping to univocal portions of each HERV-W locus; use of individual HERV-W loci sequences as a query and for the study of recombination events possibly occurring i*n vitro* during amplification protocols
Analysis of single HERV-W genes expression	No information about the full-length sequence expression, the LTR residual activity and the genomic context of insertion	Link of the HERV-W gene transcripts to the locus of origin, analysis of LTR presence and structural preservation (e.g. conservation of promoters, enhancers, transcription factor binding sites), evaluation of the HERV-W co-localization and the possible interplay with host genes
Unknown epigenetics status	Difficult interpretation of HERV-W expression cause/effect relationships	Identification of individual transcribed loci and evaluation of DNA methylation and non-coding RNAs networking, comparison of the latters between healthy and diseased (especially in those disorders affected by an altered epigenetic control, e.g. cancer and autoimmunity)
Unknown basal expression activity in healthy conditions	No reliable evaluation of altered expression in diseased contexts	Characterization of the individual HERV-W loci basal expression in healthy tissues, inclusion of paired case-control samples (optimally diseased and adjacent-healthy tissue from the same individual)
Low consideration of truncated elements	Poor knowledge regarding the potential effects of processed pseudogenes and solitary LTRs	High-throughput evaluation of truncated elements localization (insertional mutagenesis, proximity of host genes) and residual transcriptional/regulatory activity (effects of LTR conserved sites, expression of truncated RNA/proteins with functional significance), analysis of eventual unfixed *de novo* L1-mediated retrotransposition events
Insufficient amount and characterization of samples	Low statistical significance of the data, inclusion of confounding factors	Use of statistically significant samples, inclusion of healthy controls, eventual inclusion of different pathological controls, detailed characterization of individuals healthy status and behavioral components
Low consideration of individual and environmental variables	Low statistical significance of the data, inclusion of confounding factors	Analysis of eventual HERV-W polymorphisms/allelic variants among human population and their association to ethnicity, sex, age and healthy status
